# Assessment of Functioning in Older Adults Hospitalized in Long-Term Care in Portugal: Analysis of a Big Data

**DOI:** 10.3389/fmed.2022.780364

**Published:** 2022-03-15

**Authors:** Ana Ramos, César Fonseca, Lara Pinho, Manuel Lopes, Rui Brites, Adriana Henriques

**Affiliations:** ^1^Nursing Research, Innovation and Development Centre of Lisbon (CIDNUR), Nursing School of Lisbon (ESEL), Lisboa, Portugal; ^2^Escola Superior de Enfermagem de São João de Deus, Universidade de Évora, Évora, Portugal; ^3^Comprehensive Health Research Centre (CHRC), Universidade de Évora, Évora, Portugal; ^4^Instituto Superior de Economia e Gestão, Universidade de Lisboa, Lisboa, Portugal; ^5^Faculdade de Medicina, Instituto de Saúde Ambiental (ISAMB), Universidade de Lisboa, Lisboa, Portugal

**Keywords:** functioning, older adults, ICF, long-term care, self-care

## Abstract

**Background:**

Functioning assessment is a key tool for health professionals to characterize the person's degree of dependence and plan care.

**Objectives:**

The objectives were: (1) know the functioning components of older adults hospitalized in the National Network of Continuous Integrated Health Care (NNCIHC) in Portugal; and (2) compare the conceptual frameworks used in this network with the International Classification of Functioning, Disability and Health (ICF).

**Methods:**

A longitudinal retrospective study is made with 171,414 individuals aged 65 years and over. The Principal Components Analysis (PCA) was realized to reduce the number of variables, previously suggested by a scoping review, about the concepts that characterize the functionality. Then, a consensus meeting was held, where the items were matched with the ICF.

**Results:**

The average age of the sample is 80.17 years old (SD = 7.383), predominantly female (59%), without a spouse (54%), and with <6 years of education (56.4%). Four concepts were grouped: mobility, life daily activities, instrumental activities, and cognitive status that demonstrated good internal consistency. Most items correspond to ICF, except for the item “taking medication.”

**Conclusion:**

Theoretical and conceptual similarities support the use of instruments based on the ICF in Portugal's healthcare network. We suggest that ICF also encompasses a specific dimension related to medication management, given its importance for people's health.

## Introduction

The aging of the population poses a growing challenge on a global scale in regard to better respond to the needs of the older adults, particularly in terms of health care. With older age, there is an increased risk of developing chronic and degenerative diseases, which represent more than 50% of the global disease burden, with profound implications for independence and the use of health care and services (1–3). In addition, death from chronic diseases has been increasing over time, rising from 67% of deaths worldwide in 2010 to 74% in 2019 (1). In Portugal, a recent study with people over 65 years old institutionalized or supported in day centers found that 68.2% had multimorbidity, that is, they suffered from more than one chronic disease (4). A recent study concluded, in a cross-sectional analysis, that people between 60 and 69 years old who suffer from three or more diseases fit into a complex morbidity profile, which over the years refers to those who develop a severe disability in carrying out activities of daily living and have a moderate risk of mortality (5). Moreover, multimorbidity significantly increases the risk of dependence when combined with conditions that affect cognitive and mental status (6, 7).

Thus, it is to be expected that older adults with multimorbidity have a higher risk of becoming dependent. Dependence can be defined as the inability to satisfy one or more needs, essential to the maintenance of life and well-being, without a supplementary or total action on the part of another (8, 9). As such, it is a product of the combination of impairment and need, which means that it is not just a loss of aptitude, faculty, or competence to perform one or more activities. The level of dependence varies, as it incurs the degree and type of disability. It can be permanent or temporary, which means that it can be prevented, reduced, or reversed, if there is an appropriate environment and assistance (10). Dependence is not necessarily restricted to self-care, but it is in this domain that it gains special importance for individuals and is particularly sensitive for health professionals. For the WHO, self-care can be understood as an ability for individuals, families, and communities to promote and maintain health, prevent disease and deal with dependence and disability with or without the support of health professionals (11).

Health-care professionals play a key role in promoting self-care for the person with some degree of dependency. Functioning assessment is a key tool for health professionals to characterize the person's degree of dependence and plan care, being a useful tool for the assessment of health outcomes (12). To this end, the WHO developed the International Classification of Functioning, Disability and Health (ICF) in a more comprehensive attempt to classify health concepts (13, 14). The ICF is a classification system, based on an integrative biopsychosocial health model, addressing functioning and disability (14). The ICF is composed of four constructs: Body Functions (b); Body Structure (s); Activity and Participation (d), and Environmental Factors (e). Each construct of ICF is arranged in a hierarchy (chapter, second, third, and fourth level domains), e.g., Chapter 2: Sensory Functions and Pain (b2); Second level: Seeing Functions (b210); Third level: Quality of vision (b2102); and Fourth level: Color vision (b21021) (14). The ICF is used internationally to describe health and functioning, and some of the health instruments have been mapped (15). Providing care with a high level of excellence is a priority in health systems, for the provision of efficient and effective services that result in ideal results for people, especially for dependent older adults, as they are more exposed to vulnerability (16, 17).

### National Network of Continuous Integrated Health Care (NNCIHC)

The increase in situations of dependence in self-care and the need to reduce the length of hospital stay posed new challenges to health teams and families, related to the preparation for discharge. To meet this emerging need, it was created in 2006 in Portugal, the National Network of Continuous Integrated Health Care (NNCIHC) (18). Its mission is to ensure the promotion of the continuity of care in an integrated manner to people in a situation of dependency, at any age, who need continued health care and social support, of a preventive, rehabilitative, or palliative nature, provided through a unit of inpatients or outpatient clinics and hospitals and domiciliary teams, comprising a set of public and private institutions (Decree-Law no. 101/2006, of June 6). This network has a few key characteristics that are important to highlight: (1) have a dual-advice, the Ministry of Health, and the Ministry of Labor and Social Solidarity, combining health care with social care; (2) focuses on dependency and functioning gains; (3) aims at the integration and continuity of care.

These inpatient units constitute three types of networks: Convalescence, Medium Duration and Rehabilitation, and Long-Term and Maintenance. The Convalescent Units have the objective of clinical and functional stabilization, being more advisable for people recovering from an acute process or decompensation of a chronic disease, with great recovery potential with a predictable stay of up to 30 consecutive days. The Medium Duration and Rehabilitation Units are designed for transitory situations, where, for the promotion of rehabilitation, autonomy, and control of the acute or chronic process, there is a need for hospitalization ranging from 30 to 90 days. The Long-Term and Maintenance Units are intended to ensure care that prevents and/or delays the exacerbation of the dependency condition, with a likely hospital stay of more than 90 days. This time frame is extended given that care is envisioned for people with chronic diseases of slow evolution and with a high degree of complexity, which cannot and is not advised to be provided at home (Decree-Law no. 101/2006). Before admission to the Network, people are submitted to a multidisciplinary assessment and, according to their recovery potential, they are placed in a certain Inpatient Unit. Since its creation and up to 2017, an integrated assessment, based on multiple variables, was used to assess the functioning of its users. However, there is a lack of evidence about the concepts that permit to evaluate the functional capacity of older people, subsequently, to understand the correspondence of these items with the domains of the ICF. To improve the evaluation of functionality, the NNCIHC implemented, in February of 2017, a framework for developing instruments: the ICF, to allow for a more rigorous definition of outcome indicators and international comparability. The data presented in the database prior to this date included various variables about the health conditions and level of functionality. Several studies report insufficient information on the performance and results of the NNCIHC in self-care capacity (10, 19, 20) so it is relevant to analyze its impact, until the introduction of a new measurement instrument.

Mobility capacity: walking, the performance of basic life activities and instruments, and cognitive status are equally important dimensions in self-care, in long-term care. Their relationship has been studied, and they are also facilitators or inhibitors of self-care capacity (21). In this way, we can analyze the data carefully, and understand the evolution of the functioning of the inpatients of the NNCIHC in further studies.

In this sense, the objectives of this research were: (1) know the functioning components of older adults hospitalized in the NNCIHC in Portugal; and (2) compare the conceptual frameworks used in this network with the ICF.

## Materials and Methods

### Type of Study and Sample

A longitudinal retrospective study is made with a sample of 171,414 older adults, aged 65 years and over and hospitalized in health units belonging to the NNCIHC. The Long-Term and Maintenance Units were the ones with the most hospitalized people (34.7%; *N* = 59,516), followed by the Medium Term and Rehabilitation Units (34.4%; *N* = 59,013) and, finally, the Convalescent Units (30.9%; *N* = 52,885).

### Collect Data/Procedures

After authorization from the National Data Protection Commission and the Ethics Committee, the informatics services of the NNCIHC provided the data without information that would allow the identification of patients. So, data were obtained by analyzing the records of health professionals, mostly nurses, on the portal of the NNCIHC, called Network GestCare, from 2010 to 2017.

After collecting data, we performed the following steps:

1) Analysis of the functioning components of older adults hospitalized in the NNCIHC in Portugal.
2) Compare the conceptual frameworks used in this network with the ICF.

### A New ICF-Based Instrument

In the Network GestCare set of information from the electronic health, the record is available, based on several items of validated international scales. The variables present included sociodemographic characteristics, health complaints, nutritional status, falls, mobility, physical autonomy based on Katz Index of Independence in Activities of Daily Living (22), instrumental autonomy on Instrumental Activities of Daily Living (23), emotional complaints and cognitive state, based on the Mini-Mental State Examination (24). The data received from the GestCare Network were the results of the assessment of patients in the units using the ICF-based instrument. This instrument is not validated yet, so we intend to analyze some of its variables and test its reliability.

### Data Analysis

Descriptive statistics were used to analyze the sociodemographic characteristics by type of care.

To address objective 1: assess to the variables that characterize the functioning concepts of older adults hospitalized in the national network of integrated continuous health care in Portugal; we conducted the following steps:

(a) Tested the construction of the concepts, a PCA was performed, as it is a multivariate exploratory analysis technique that transforms a set of correlated variables into a smaller set of independent variables. When studying a database with many information and variables, PCA was performed as one of the most common data reduction methods. It is a multivariate exploratory analysis technique that transforms a set of variables correlated with each other into a smaller set of independent variables, with linear combinations of the original variables, called main components (25). We can reduce it to a total of 22 variables (indicators) into 4 variables (concepts), to simplify the process of analysis.
(b) Analyzed the importance of the different variables to the global concept. The Kaiser–Meyer–Olkin (KMO) was analyzed, and an examination of the commonalities (*h*^2^) was performed to identify which variables were more or less important, in terms of the variance explained for the analysis of the components. A value of 0.5 was defined as the acceptable lower limit for the inclusion of variables. Bartlett's test of sphericity presented a *p* < 0.001 in all concepts, so we reject the null hypothesis and conclude that the variables are significantly correlated.
(c) Calculated Cronbach's α to analyze the internal consistency of the items and each of the domains (26). According to Murteira (27), missing's in big data, characterized by their high number of data, are irrelevant, so they were not included in the statistical analysis.

To address objective 2: compare the conceptual frameworks used in this network with the ICF, we conducted the following steps:

Step 1) To match the variables of self-care with the ICF items, the focus group method was used, and an evaluation was performed by experts in the field. The criteria for belonging to the focus group were as follows: experience in functionality assessment and in-depth knowledge of the ICF. The focus group consisted of 5 participants (one physician, two nurses, and two physical therapists) and it was moderated by one of the group's researchers.
Step 2) Analyzed the final concepts and their compatibility to the ICF, as in a previous study (15), the concepts and the relationship to the ICF items were discussed. All had more than 80% agreement, so there was no need to resolve convergences. We have taken the ICF linking rules into consideration in this process (28, 29).

## Results

### Sociodemographic Characteristics

The sample consisted of 171,414 older adults, with an average age of 80.17 years (SD = 7.383), between 65 and 109 years. Most of the sample is female (59.0%), does not have a spouse, given that 37.2% have the marital status of widowhood, 13.0% are single and 3.8% are divorced/separated and have <6 years of schooling (56.4%) (Table 1). The number of individuals may be smaller in some of the analyzed variables, triggered by the existence of missing's (missing values).

**Table 1:** Sociodemographic characterization of the sample (*N* = 171,414).

**Sociodemographic** **variables**	**Global** ***n* (%)**	**Convalescent units** ***n* (%)**	**Medium term and rehabilitation units** ***n* (%)**	**Long term and maintenance units** ***n* (%)**
**Age**				
65–74	40,409 (23.6)	15,320 (29.0)	14,498 (24.6)	10,591 (17.8)
75–84	79,899 (46.6)	24,987 (47.2)	28,414 (48.1)	26,498 (44.5)
≥ 85	51,106 (29.6)	12,578 (23.8)	16,101 (27.3)	22,427 (37.7)
**Sex**				
Female	101,150 (59.0)	32,535 (61.5)	34,215 (58.0)	34,400 (57.8)
Male	70,264 (41.0)	20,350 (38.5)	24,798 (42.0)	25,116 (42.0)
**Marital Status**				
Single	19,945 (11.6)	6,193 (11.7)	6,332 (10.7)	7,420 (12.5)
Married	70,491 (41.2)	20,883 (39.5)	24,832 (42.1)	24,776 (41.6)
Divorced	5,782 (3.4)	2,018 (3.8)	2,166 (3.7)	1,598 (2.7)
Widow	57,076 (33.3)	17,578 (33.2)	19,510 (33.1)	19,988 (33.6)
Unknown	18,120 (0.2)	109 (0.2)	90 (0.2)	97 (0.2)
Missing	17,824(10.4)	6,104 (11.5)	6,083 (10,3)	5,637 (9.5)
**Education (years)**				
No education	33,596 (19.6)	8,244 (15.6)	10,933 (18,5)	14,419 (24.2)
1 to 6	51,879 (30.3)	15,802 (29.9)	18,564 (31,5)	17,513 (29.4)
7 to 12	3,395 (2.0)	1,087 (2.1)	1,346 (2,3)	962 (1.6)
≥ 13	3,164 (1.8)	1,047 (20)	1,248 (2,1)	869 (1.5)
Missing	79,380 (46.3)	26,705 (50.5)	26,922 (45.6)	25,753 (43.3)
**Portugal Region**				
Alentejo	15,165 (8.8)	4,913 (9.3)	4,668 (7.9)	5,584 (9.4)
Algarve	8,937 (5.2)	3,659 (6.9)	2,761 (4.7)	2,517 (4.2)
Center	42,802 (25.0)	11,937 (22.6)	15,233 (25.8)	15,632 (26.3)
Lisbon and Vale do Tejo	45,872 (26.8)	11,362 (21,5)	18,663 (31.6)	15,847 (26.6)
North	49,565 (28.9.5)	18,805 (35.6)	14,778 (25.0)	15,982 (26.9)
Missing	18,146 (5.3)	2,209 (4.2)	2,910 (4.9)	3,954 (6.6)

### Reliability Analysis

The factors or components of self-care were selected according to the theoretical framework, a scoping review carried out previously (21) and with the objective of the study. These components include (1) mobility: walking; (2) Activities of Daily Living (ADL); (3) Instrumental Activities of Daily Living (IADL); and (4) cognitive state: orientation in time and space. The sample consisted of 159,084 assessments of the health status of the elderly.

The analysis of the commonalities (h^2^) is important to identify which variables are more or less important, in terms of the explained variance for the analysis of the components. In view of the literature (26), it was considered that variables with commonality lower than 0.5 have low explanatory power in the variance of the components, being defined as the acceptable lower limit for the inclusion of variables. It was found that the KMO statistic is an indicator of partial correlations between high variables, namely in the concept of basic (KMO = 0.885) and instrumental (KMO = 0.917) activities. The concept related to mobility: walking (KMO = 0.743) and cognitive status (KMO = 0.593) assume lower values, probably due to the smaller number of items, respectively three and two, as shown in Table 2. Bartlett's test of sphericity presented a p < 0.001 in all concepts, so we concluded that the variables are significantly correlated.

**Table 2:** Analysis of the main components and the alpha coefficients (*N* = 159,084).

**Indicators**	**Concepts**				
	**Mobility—walking**				
	**Component matrix**				***h^**2**^***
Walk in the street	**0.932**				0.869
Use stairs	**0.893**				0.798
Walk at home/inside buildings	**0.895**				0.800
	**Activities of daily living (ADL)**				
Use toilet, potty and/ or urinal (use, clean up, clothes, flush)			**0.906**		0.822
Lay down/ Get up from the bed (move, transfer, walk)			**0.905**		0.818
Sit down/ get up from chairs (move, transfer, walk)			**0.902**		0.814
Get dressed/take off clothing (choose, prepare, and get dressed)			**0.888**		0.789
Wash up/take a shower (go in/out, be, clean up)			**0.852**		0.725
Control feces			**0.818**		0.668
Control of urine			**0.808**		0.652
Feed/eat (serve, prepare food, eat)			**0.769**		0.592
**Instrumental activities of daily living (IADL)**					
Meal preparations			**0.911**		0.880
Laundry			**0.910**		0.920
Housework			**0.909**		0.925
Shop			**0.897**		0.847
Use transports			**0.857**		0.754
Manage money			**0.809**		0.818
Take medication			**0.749**		0.787
Use of telephone			**0.681**		0.785
	**Cognitive state—Time and space orientation**				
Time and Space Orientation (global assessment)				**0.995**	0.990
Space Orientation				**0.960**	0.921
Time Orientation				**0.970**	0.941
***Cronbach's Alpha***	0.889	0.938	0.922	0.974	0.951
*Kaiser*- Meyer-Olkin	0.743	0.885	0.917	0.593	
Bartlett's Test of Sphericity	379,877.629	6,645,154.241	1,457,746.534	3,811,525.473	
	3	28	28	3	
	0.000	0.000	0.000	0.000	

*The meaning of bold values is most relevant information in this table*.

The overall Cronbach's α (for the 22 items) had a value of 0.95, demonstrating excellent internal consistency. Regarding the Cronbach's α of the concepts, the cognitive state had the highest alpha (α = 0.97), followed by the concept self-care: basic activities (α = 0.94), the concept self-care: instrumental activities (α = 0.92), and mobility: walking (α = 0.89).

### Corresponding ICF Concepts

From the similarity analysis of the concept mapping obtained with the ICF, it is possible to verify that there is a correspondence between the cognitive state: orientation in time and space with the functions of orientation (b114), orientation in relation to time (b1140) and orientation in relation to place (b1141), inserted in the mental functions.

The concept of mobility, more particularly walking on the street, fits into walking long distances (d501), just as walking on stairs can translate into going up/down (d4551), just as walking at home/inside buildings is similar to walking short distances (d500).

Relatively, the activities of life that involve lying down/getting out of bed and sitting down or getting up from chairs fall under the ICF in changing the basic position of the body (d410). Using the toilet, potty, and/or urinal, as well as controlling the sphincters, its association in the ICF with care related to the excretion processes is suggested (d530). Dressing and undressing with similar semantics fits into dressing (d540), as well as washing/bathing with washing (d510) and eating/eating with eating (d550) and drinking (d650).

Instrumental life activities that include using transport fit in the ICF to the use of transports (d470); using the phone when using communication devices (d3600); housework chores to performing housework chores (d640); make purchases to buy (d6200) or the acquisition of goods and services (d620) and manage money for basic economic transactions (d860). Preparing meals uses exactly the same terminology (d630) as washing clothes (d6400).

More detailed information is shown in Table 3.

**Table 3:** Instrument's items x aligned with the corresponding ICF (*International Classification of Functioning, Disability and Health*) concepts.

**ICF component**	**ICF chapter**	**ICF chapter concepts**	**Integrated assessment instrument**		**ICF correspondence**
			**Concept**	**Items**	
Body functions (B)	B1	Mental functions	Cognitive state - time and space orientation	Time and space orientation (global assessment)	b114 Orientation functions
				Time Orientation	b1140 Orientation to time
				Space Orientation	b1144 Orientation to space
Activities and participation (D)	D4	Mobility	Mobility: walk	Walk in the street	d450 Walking: d4501 walking short or long distances
				Use stairs	d455 Moving around: d4551 Going up and down stairs
				Walk at home/inside buildings	d450 Walking: d4500 Walking short distances
			Instrumental activities	Use transports	d470 Using transportation
			Basic activities	Lay down/ Get up from the bed (move, transfer, walk)	d410 Changing basic body position: d4100 Laying down; d4104 Standing
				Sit down/ get up from chairs (move, transfer, walk)	d410 Changing basic body position: d4103 Sitting; d4104 Standing
	D5	Self-care	Basic activities	Use toilet, potty and/ or urinal (use, clean up, clothes, flush)	d530 Toileting
				Get dressed/take off clothing (choose, prepare and get dressed)	d540 Dressing: d5400 Putting on clothes; d5401 taking off clothes; d5404 Choosing appropriate clothing
				Wash up/take a shower (go in/out, be, clean up)	d510 Washing oneself: d5101 Washing whole body
				Control feces	d530 Toileting: d5301 Regulating defecation
				Control of urine	d530 Toileting: d5300 Regulating urination
				Feed/eat (serve, prepare food, eat)	d550 Eating: d560 Drinking
	D3	Communication		Use of telephone	d360 Using communication devices and techniques: d3600 Using telecommunication devices
	D6	Domestic life	Instrumental activities	Meal preparations	d630 Preparing meals
				Housework	d640 Doing housework
				Laundry	d640 Doing housework: d6400 Washing and drying clothes and garments
				Shop	d620 Acquisition of good and services: d6200 Shopping
	D8	Main life areas		Manage money	d860 Basic economic transactions
				Take medication	

## Discussion

This study has two main objectives, first, we intend to know the functioning components of older adults hospitalized in the NNCIHC in Portugal; and second, we aimed to compare the conceptual frameworks used in this network with ICF.

Regarding the sociodemographic profile, older adults between 65 and 74 years were hospitalized in greater numbers in Convalescence Units (29.0%) and less in Long-Term Units (17.8%). Older people (85 or over) were the most frequent in Long-Term and Maintenance Units (37.7%). While, in the Medium Duration and Rehabilitation groups, individuals aged between 75 and 84 years predominated (48.1%). Fluctuations in the three typologies were little accentuated in marital status, highlighting the absence of a spouse/sentimental partner. There was a great predominance of widowed and single/divorced women, with a greater number of married men. Older adults who did not attend school (42.7%) were more concentrated in Long-Term and Maintenance Units, probably related to the older age of people.

### Integrated Assessment Instrument and ICF Concepts

The principal components that described the functional capacity, used by health professionals from the NNCIHC, had excellent internal consistency. In the matrix of components, most of the very high correlation coefficients (> 0.8) are detected, which reinforces the results found for the sphericity test and the KMO measure.

So, regarding our second goal, it was possible to obtain correspondence with the ICF in almost all items present in the Integrated Assessment Instrument, with the exception of the item “Taking medications,” as this concept is not in the ICF. However, we can fit it into the Self-Care dimension of the ICF. The item “Maintaining one's health,” according to the ICF, refers to caring for oneself by doing what is required to look after one's health (11). We consider that taking medication is an activity that is related to taking care of one's own health. Although it does not specify the taking of the medication, it is the category where the item “Taking medications” best fits. However, the definition of the concept itself does not clearly address medication management. Furthermore, we consider that this activity is extremely relevant for the maintenance of health, especially in the management of one or more chronic diseases, and should be considered as a unique dimension of self-care. A scoping review that evaluated interventions to improve self-care in patients with a chronic condition reported that maintenance behaviors focused primarily on physical activity (70%), food intake (59%), and medication intake (52%). However, monitoring of medication intake was rarely included (9%) and was mainly done in studies with patients with heart failure (15%) and asthma (20%) (30).

### Components of Self-Care

This study highlighted self-care as the main aspect of functioning that affects the lives of older adults. As reflected in the results, we developed concepts of functioning, which in turn interfere with self-care: mobility; basic activities; instrumental activities; and cognitive status. Self-care can be considered a broad concept that encompasses the other concepts (31), so there is a need for a systematic assessment of geriatric conditions (32). There are several factors that can influence self-care, such as taking sedative or psychotropic medications, cognitive impairment, depression, and a history of falling (33, 34). In this study, patients hospitalized in the NNCIHC present changes in self-care due to impairments in functioning caused either by disorders or temporary loss of function due to a specific situation, such as a fall.

The changes in self-care are related to the following disease-related factors, reported by Palmer (35) multimorbidity (difficulty in integrating self-care in all conditions); and life events that interact with the disease to interfere with healthy behavior. In the case of our sample, life events are diverse: dependence on life activities, need for teaching the person/informal caregiver, rehabilitation, post-surgical care, and pressure ulcers. It is also highlighted as a reason for hospitalization, the management of the caregiver's therapeutic regimen and rest, with the need for continuous integrated care.

The care models should describe more specific individual levels of activities and processes (36). A recent literature review defines a self-care model that encompasses functioning components related to body functions, cognition, and emotional functions, highlighting the importance of self-management of life activities (37).

### Self-Care and the Person-Centered Care

Since people have specific individual and environmental characteristics, more and more person-centered care should be chosen in the healthcare units (38).

Person-centered care has increasingly demonstrated effectiveness in health outcomes, and functioning assessment is the key to promote self-care, as it allows for the assessment of the evolution of functional status and planning of care accordingly. Studies demonstrate the importance of person-centered care in increasing the activation of hospitalized older adults, enhancing their capacity for selfcare (30).

To implement person-centered care, we consider that there are other concepts that should be introduced in the evaluation of the functioning of older adults admitted to the NNCIHC, such as communication and environmental factors, such as support from networks (family, friends, other providers), given their interference in self-care. The Elderly Nursing Core Set (ENCS) is a recently constructed and validated instrument for the Portuguese population, which is already being used in research studies (4, 39) and addresses the concepts: self-care, learning, and mental functions, communication and social relationships. It was built using the ICF concepts and some of them coincide with those presented here. It is, however, organized differently and considers a fundamental concept, the environmental factors, which are not used in the NNCIHC. Several studies have emphasized the importance of environmental factors, such as social support. For example, in the USA, there are Acute Care Units for the Older Adults (ACE) which are based on a person-centered care model that objectives to prevent the loss of independence of the patient. In these units, functional status is assessed on admission and throughout hospitalization, considering the same domains: ADL, mobility, mood/affect, cognition, living situation, social support, and nutritional status (35). In a study developed with seniors over the age of 80, the results suggest that affectionate relationships are necessary for maximal adaptation in old age (40). Besides that, several studies have found that social support can have a significant positive effect on recovery from surgery, total mortality rates, healthcare utilization, depression, teenage pregnancy, and several other conditions. Most of these studies highlight the ability of social support to moderate or buffer the impact of psychosocial stress on physical and mental health (41, 42).

### Limitations

As limitations to this study, we highlight the fact that we only have data until 2017. As of the year 2018, the data in the Integrated Continuous Health Care Network in Portugal have changed and it is for this reason that we only have data up to 2017.

## Conclusion

Theoretical and conceptual similarities support the use of instruments based on the ICF in Portugal's national integrated continuing health care network. The mobility concepts: walking; life activities; instrumental activities and cognitive status: orientation in time and space, similarly to the ICF, contribute to measuring and understanding people's health status, self-care capacity, and functioning, particularly in the context of long-term care (43–46). We suggest that ICF also encompasses a specific dimension related to medication management, given its importance for people's health.

## Data Availability Statement

The original contributions presented in the study are included in the article/supplementary material, further inquiries can be directed to the corresponding author.

## Ethics Statement

The study was conducted according to the guidelines of the Declaration of Helsinki, and approved by the Ethics Committee of Scientific Research in the Areas of Human Health and Welfare of the University of Évora (report number 17036 and date of approval April 26, 2017). Written informed consent for participation was not required for this study in accordance with the national legislation and the institutional requirements.

## Author Contributions

AR contributed to conceptualization. AR, CF, RB, and LP contributed to methodology. AR and RB contributed to formal analysis and software. CF, ML, and AH contributed to validation, writing, reviewing, and editing. CF and ML contributed to investigation, project administration, and funding acquisition. AR and LP contributed to writing original draft preparation and resources. RB contributed to data curation. All authors have read and agreed to the published version of the manuscript.

## Funding

This research was funded by FEDER. Programa Interreg VA España-Portugal (POCTEP), Grant Number 0499_4IE_PLUS_4_E. This research received external funding for the PhD scholarship from the Foundation for Science and Technology, Portugal (to AR).

## Conflict of Interest

The authors declare that the research was conducted in the absence of any commercial or financial relationships that could be construed as a potential conflict of interest.

## Publisher's Note

All claims expressed in this article are solely those of the authors and do not necessarily represent those of their affiliated organizations, or those of the publisher, the editors and the reviewers. Any product that may be evaluated in this article, or claim that may be made by its manufacturer, is not guaranteed or endorsed by the publisher.
